# Salivary Gland Stem Cells Age Prematurely in Primary Sjögren's Syndrome

**DOI:** 10.1002/art.40659

**Published:** 2018-12-27

**Authors:** Sarah Pringle, Xiaoyan Wang, Gwenny M. P. J. Verstappen, Janneke H. Terpstra, Clarence K. Zhang, Aiqing He, Vishal Patel, Rhiannon E. Jones, Duncan M. Baird, Fred K. L. Spijkervet, Arjan Vissink, Hendrika Bootsma, Robert P. Coppes, Frans G. M. Kroese

**Affiliations:** ^1^ University of Groningen and University Medical Center Groningen The Netherlands; ^2^ Bristol‐Myers Squibb Lawrence Township New Jersey; ^3^ Bristol‐Myers Squibb Pennington New Jersey; ^4^ Cardiff University School of Medicine Cardiff UK

## Abstract

**Objective:**

A major characteristic of the autoimmune disease primary Sjögren's syndrome (SS) is salivary gland (SG) hypofunction. The inability of resident SG stem cells (SGSCs) to maintain homeostasis and saliva production has never been explained and limits our comprehension of mechanisms underlying primary SS. The present study was undertaken to investigate the role of salivary gland stem cells in hyposalivation in primary SS.

**Methods:**

SGSCs were isolated from parotid biopsy samples from controls and patients classified as having primary SS or incomplete primary SS, according to the American College of Rheumatology/European League Against Rheumatism criteria. Self‐renewal and differentiation assays were used to determine SGSC regenerative potential, RNA was extracted for sequencing analysis, single telomere length analysis was conducted to determine telomere length, and frozen tissue samples were used for immunohistochemical analysis.

**Results:**

SGSCs isolated from primary SS parotid gland biopsy samples were regeneratively inferior to healthy control specimens. We demonstrated that SGSCs from samples from patients with primary SS are not only lower in number and less able to differentiate, but are likely to be senescent, as revealed by telomere length analysis, RNA sequencing, and immunostaining. We further found that SGSCs exposed to primary SS–associated proinflammatory cytokines we induced to proliferate, express senescence‐associated genes, and subsequently differentiate into intercalated duct cells. We also localized p16+ senescent cells to the intercalated ducts in primary SS SG tissue, suggesting a block in SGSC differentiation into acinar cells.

**Conclusion:**

This study represents the first characterization of SGSCs in primary SS, and also the first demonstration of a linkage between an autoimmune disease and a parenchymal premature‐aging phenotype. The knowledge garnered in this study indicates that disease‐modifying antirheumatic drugs used to treat primary SS are not likely to restore saliva production, and should be supplemented with fresh SGSCs to recover saliva production.

## Introduction

Between 0.4 and 3.1 million people in the US have been diagnosed as having the autoimmune disease primary Sjögren's syndrome (SS) 1. Presenting clinically predominantly in women (9:1 ratio), primary SS is a multifaceted syndrome most often associated with production of autoantibodies (SSA/Ro and SSB/La), infiltration of the salivary glands (SGs) with lymphocytes, and hyposalivation (reduced secretory function of the SGs). Other symptoms may include neurologic involvement, lung symptoms, and chronic fatigue. Lymphocytic infiltration of SGs is characterized and measured clinically by the presence of immune foci, defined as a gathering of >50 lymphocytes in the SGs, associated with the striated ducts. The periductal infiltrates may evolve into ectopic lymphoid tissue harboring germinal centers (sites of memory B cell formation). In addition, the relative number of IgA plasma cells decreases, in parallel with glandular dominance of IgG‐producing plasma cells. Mucosa‐associated lymphoid tissue lymphomas are also frequently observed in the SG tissue of patients with primary SS. These features of salivary gland infiltration reflect the B cell–dominated phenotype of primary SS 2. Given these characteristic lymphocytic infiltrates, the logical conclusion is that lymphocytic infiltration of SGs is the causative factor underlying hyposalivation. However, recent detailed research has clearly demonstrated that the correlation between salivary flow and degree of inflammation is weak 3, 4, 5, 6, 7, 8, 9, 10, 11.

In healthy SGs, homeostasis is maintained by proliferation and differentiation of tissue‐resident SGSCs. According to one of the prevailing views in the field, these cells reside in striated ducts, from where they differentiate first toward intercalated ducts and subsequently to acinar cells 12, 13, 14, 15, 16. Other studies have identified progenitor cell populations within the acinar cell subset, and, alternatively, have suggested that acinar cells themselves are capable of maintaining SG homeostasis through self‐replication 17, 18. Regardless of the predominant viewpoints, the apparent lack of ability of SGSCs to maintain SG homeostasis in primary SS has never been explained, and likely contributes to hyposalivation development in primary SS. In this study, we used protocols for SGSC isolation recently developed by us and others (12–14,16,19) to probe the involvement of SGSCs in primary SS. We demonstrate that SGSCs in primary SS are likely to be senescent, a phenotype that may be induced by exposure to primary SS–associated proinflammatory cytokines.

## Patients and methods

#### Source of SG tissue

For healthy control specimens, biopsy samples of parotid SG tissue were obtained from donors (after informed consent and institutional review board [IRB] approval) who were treated for a squamous cell carcinoma of the oral cavity. In these patients, an elective head and neck dissection procedure was performed. During this procedure, a parotid SG is exposed and removed. This tissue does not contain malignant cells, as oral squamous cell carcinoma does not disseminate to the parotid SG. To obtain study samples from patients with primary and incomplete primary SS, specimens were obtained during the routine diagnostic evaluation for primary SS. Patients were classified as having primary SS if they fulfilled the 2016 American College of Rheumatology (ACR)/European League Against Rheumatism (EULAR) criteria 20. Patients classified as having incomplete primary SS did not fulfill these criteria and were not taking hyposalivation‐inducing medication, but they did demonstrate either objective symptoms of dry mouth or SSA autoantibody presence. IRB approval was obtained, and all patients with primary SS and incomplete primary SS provided informed consent (METc 2016/010).

#### Stem cell isolation

Parotid SG biopsy samples harvested from oral squamous cell cancer patients with healthy parotid gland tissue and samples obtained from patients with primary SS and incomplete primary SS after surgery were processed in Hanks’ balanced salt solution (HBSS) containing 1% bovine serum albumin (BSA; Invitrogen). Biopsy samples were digested mechanically using a gentleMACS dissociator (Miltenyi Biotec) or manually with scissors and simultaneously digested in HBSS/1% BSA buffer containing 0.63 mg/ml type II collagenase (Invitrogen) and 0.5 mg/ml hyaluronidase (Sigma‐Aldrich), as well as calcium chloride at a final concentration of 6.25 m*M*, for 30 minutes at 37°C. Forty milligrams of tissue was processed per 1 ml buffer volume; total volume was adjusted according to biopsy sample weight. Digested cells were collected by centrifugation, washed twice in HBSS/1% BSA solution, and passed through 100‐μm cell strainers (BD Biosciences).

The resultant cell suspensions were collected again by centrifugation and resuspended in SGSC medium consisting of 40% Dulbecco's modified Eagle's medium/F‐12 medium, penicillin/streptomycin antibiotics (Invitrogen), Glutamax (Invitrogen), 50% Wnt‐3a–conditioned medium, 10% R‐spondin–conditioned medium (derived from the RSPO1 cell line; AMSBIO), 20 ng/ml epidermal growth factor (Sigma‐Aldrich), 20 ng/ml fibroblast growth factor 2 (Sigma‐Aldrich), N2 (Invitrogen), 10 mg/ml insulin (Sigma‐Aldrich), 1 m*M* dexamethasone (Sigma‐Aldrich), 10 μ*M* Rho kinase inhibitor (Abcam), 5 μ*M* transforming growth factor β inhibitor (catalog no. A8301; ToCris Bioscience), and 12.5 ng/ml Noggin (PeproTech). A total of 800,000 primary isolate cells were resuspended in 25 μl of SGSC medium combined with 50 μl of basement membrane Matrigel (BD Biosciences) and deposited in the center of 12‐well tissue culture plates. After the gels were allowed to solidify (20 minutes at 37°C), 1 ml of stem cell medium was added per well. After 3–5 days of culture, the formed primary spheres were released from Matrigel by incubation in 1 mg/ml Dispase (Sigma) (1 hour at 37°C). Primary spheres of a minimum size of 50 μ*M* were counted and used to establish primary sphere yield per milligram of biopsy material. To correct primary sphere yield for the site of biopsy, the primary sphere yields for healthy control samples and primary SS samples were multiplied by factors of 4.1 and 11.95, respectively. Multiplication factors were derived from the yield of primary spheres isolated from the SGSC‐rich area, as described by van Luijk et al 21.

#### Cytospot preparation and quantification

A total of 100 μl of cell suspension obtained after SGSC isolation was added into a cytospin funnel, after prewetting of coated microscope slides with 1% BSA/phosphate buffered saline (PBS) solution. After centrifugation at 300 revolutions per minute for 2 minutes, slides were air dried and fixed with 4% paraformaldehyde (PFA) at room temperature for 20 minutes. Hematoxylin and eosin staining was then performed according to standard protocols. The number of acinar and ductal cells was determined by capturing images of 3 areas of the cytospot per sample. The total cell number in each area was determined by counting hematoxylin‐stained nuclei. Acinar cells were identified by characteristic triangular morphology and predominant hematoxylin staining. Ductal cells were identified by heavily eosin‐stained cytoplasm. The proportion of each cell type was expressed as the percentage of total cells. For CD45+ cell quantification, cytospots were fixed as described above, then permeabilized in 100% ethanol (20 minutes at −20°C), washed in PBS, and then incubated in mouse anti‐CD45 antibody (Dako) (1 hour at room temperature), diluted 1:100 in 1% BSA containing 0.05% Tween. Following PBS washing, Alexa Fluor 488–conjugated goat anti‐mouse secondary antibody was added onto cytospots at 1:300 dilution in 1% BSA/0.05 PBS containing Tween and incubated at room temperature for 1 hour. Following final PBS washes, nuclei were counterstained with DAPI, and cytospots were visualized using a Leica 6000 Series microscope.

#### Flow cytometry and fluorescence‐activated cell sorting (FACS) of SG isolate

After isolation, cell suspensions were dispersed to single cells. Cells were immunolabeled with antibodies against the following human proteins, conjugated to fluorophores as indicated: eFluor 660–conjugated epithelial cell adhesion molecule (EpCAM) (1:20; eBioscience), phycoerythrin (PE)–Cy5–conjugated CD45 (1:50; BioLegend), BUV737‐conjugated CD19 (1:50; eBioscience), allophycocyanin (APC)–eF700–conjugated CD3 (1:50; eBioscience), PE‐Cy7–conjugated CD56 (1:50; BioLegend), APC‐eF780–conjugated CD4 (1:50; eBioscience), PE‐Cy7–CD24 (1:20; BioLegend), and fluorescein isothiocyanate –conjugated Ki‐67 (1:200; ThermoFisher Scientific). For intranuclear staining for Ki‐67, a Foxp3 Transcription Factor Buffer Set was used, according to the instructions of the manufacturer (eBioscience). Staining for K14 and smooth muscle actin (SMA) was performed in 2 steps using rabbit anti‐human K14 (1:100; Abcam) and mouse anti‐human SMA (1:100; Dako) and Alexa Fluor 647–conjugated secondary antibody (1:300). Antibodies were added in a total volume of 100 μl 0.5% BSA/PBS with 2 m*M* EDTA (staining buffer), containing a maximum of 1 million cells. Staining was performed for 20 minutes on ice. Cells were collected by centrifugation and resuspended in staining buffer for analysis with an LSRII flow cytometer (BD Bioscience).

Living–dead discrimination was performed using 80 ng/ml propidium iodide (ThermoFisher). For FACS sorting of EpCAM+ cells from SG isolate, staining was performed as described above, with the addition of 0.1*M* magnesium sulfate and 50 μg/ml DNase (both from Sigma) into cell suspension to prevent cell clumping. Collected CD45+ cells were harvested into stem cell medium collected by centrifugation and plated into Matrigel as described above. The gating strategy for flow cytometric analysis and FACS is shown in Supplementary Figure 1, available on the *Arthritis & Rheumatology* web site at http://onlinelibrary.wiley.com/doi/10.1002/art.40659/abstract.

#### Self‐renewal

Following the release of primary spheres from Matrigel as described above, cells were dispersed to form single‐cell suspensions using 0.05% trypsin–EDTA (Invitrogen), enumerated, and concentrations adjusted to 0.4 × 10^6^ cells per ml in SGSC medium. Twenty‐five microliters of this cell solution was combined with 50‐μl volumes of basement membrane Matrigel and deposited in the center of 12‐well tissue culture plates. After the Matrigel was solidified for 20 minutes at 37°C, gels were covered in stem cell medium as described above. Organoids appeared 2–3 days after seeding of single cells in Matrigel. Ten days after seeding, Matrigel was dissolved by incubation with Dispase enzyme as described above. Organoids >50 μ*M* in diameter were enumerated, cells were processed to a single‐cell suspension using 0.05% trypsin–EDTA, and cell numbers were determined. These data were used to generate the organoid formation efficiency and population doublings. Population doublings (pds) were calculated according to the following formula:

$$\text{pds}=\frac{\text{ln}2\;(\text{harvested cells/seeded cells})}{\text{ln}2}$$

Encapsulation in Matrigel was repeated to generate the next passage. This cycle was repeated 4 times (4 passages). At the end of each passage, an image of the cells was captured using an Olympus CKX53 microscope and DP2‐SAL software.

#### Mature organoid formation assay

For mature organoid formation assays, organoid cultures were supplemented with 1 μ*M* isoproterenol. Mature organoid formation was monitored over a 2‐week period.

#### RNA sequencing

Total RNA was extracted from stem cells using an Absolutely RNA Miniprep Kit (catalog no. 400800), according to the instructions of the manufacturer (Agilent). The integrity of RNA was examined with an Agilent 2100 Bioanalyzer. Subsequent sequencing was performed using a SMART‐Seq v4 Ultra Low Input RNA Kit (catalog no. 634890; Clontech) and a Nextera XT DNA Library Prep Kit (catalog no. FC‐131‐1096; Illumina) according to the instructions of the manufacturers, and prepared DNA libraries were sequenced on a HiSeq 2500 system. Data quality assessment was performed to understand the main source of variability, and differential expression analysis and visualization were performed in R (packages PVCA, EdgeR, and PHeatmap). The MetaCore pathway database was used for pathway enrichment analysis; data can be accessed via the NCBI Sequence Reads Archive (accession no. PRJNA506620).

#### Cytokine incubations with SGSCs

Cytokines were purchased as follows: human interleukin‐6 (IL‐6) (catalog no. 200‐06A; PeproTech), human interferon‐α (IFNα) (catalog no. 11100‐1; R&D Systems), and human tumor necrosis factor (TNF) (catalog no. 300‐01A; PeproTech) and reconstituted according to the instructions of the manufacturers. Dilutions for coculture with cytokines were performed in such a manner that the volume of cytokine added to the medium was always 1% of the total medium volume. The medium was refreshed 2 times within the passage (days 3 and 6), in parallel with control cultures.

#### Whole‐mount and tissue immunocytochemistry

Mature organoids were released from Matrigel using Dispase, collected in round‐bottomed 96‐well plates, and fixed in 2% PFA for 10 minutes. Frozen tissue sections were cut at a thickness of 8 μ*M* and fixed in 2% PFA for 5 minutes. Staining for all samples was performed from this point, using a Tyramide Signal Amplification Kit according to the instructions of the manufacturer (ThermoFisher). After hydrogen peroxide blocking and general blocking, primary antibodies were incubated with organoids, mature organoids, or tissue sections overnight in PBS at 4°C. Dilutions of primary antibodies used for immunostaining were as follows: rabbit anti‐human amylase (1:100) (catalog no. A2863; Sigma), rabbit anti‐human aquaporin 5, rabbit anti‐human EpCAM, mouse anti‐human IL‐6 receptor (1:100) (clone B‐R6; ThermoFisher), mouse anti‐human TNF receptor type I (clone H398;ThermoFisher), rabbit anti‐human IFNα receptor (1:100) (catalog no. 62693; Abcam), mouse anti‐human p16 (1:100) (catalog no. 54210; Abcam), and mouse anti‐human SMA (1:100) (catalog no. M0851; Dako). Nuclear counterstaining was performed with Hoechst 33342, at a 1:300 dilution from 10 mg/ml stock solution, for 10 minutes at room temperature. Immunostainings were visualized using a Leica TCS SP8 confocal laser scanning microscope and Leica Application Suite software.

#### Telomere analysis

DNA was extracted from human SGSCs using a QIAamp DNA Micro Kit (Qiagen). Single telomere length analysis (STELA) was carried out at the XpYp telomere as described previously by Capper et al 22. Briefly, 1 μ*M* Telorette 2 linker was added to 10 ng of purified genomic DNA in a final volume of 40 μl per sample. Multiple polymerase chain reactions (PCRs) were performed for each test DNA in 10‐μl volumes, incorporating 250 pg of DNA, 0.5 μ*M* telomere‐adjacent and Teltail primers, 75 m*M* Tris HCl (pH 8.8), 20 m*M* (NH_4_)_2_SO_4_, 0.01% Tween 20, 1.5 m*M* MgCl_2_, and 0.5 units of a 10:1 mixture of Taq (ABgene) and Pwo polymerase (Roche Molecular Biochemicals). The reactions were processed in a Tetrad2 Thermal Cycler (Bio‐Rad). DNA fragments were resolved by 0.5% Tris–acetate–EDTA agarose gel electrophoresis and identified by Southern hybridization with a random‐primed a‐^33^P‐labeled (PerkinElmer) TTAGGG repeat probe, together with probes specific for the 1 kb (Stratagene) and 2.5 kb (Bio‐Rad) molecular weight markers. Hybridized fragments were detected using a Typhoon FLA 9500 Phosphorimager (GE Healthcare). The molecular weights of the DNA fragments were calculated using a Phoretix 1D Quantifier (Nonlinear Dynamics).

#### Quantitative PCR (qPCR)

Total RNA was extracted from cultured cells using an RNeasy Microkit, including DNase incubation, according to the instructions of the manufacturer (Qiagen). One microgram of total RNA was reverse transcribed to complementary DNA (cDNA) using 0.5 μg of oligo(dT)_15‐18_ primers, 1.0 m*M* dNTPs, 1× Reaction Buffer, 20 units of RiboLock, and 200 units of RevertAid reverse transcriptase (all from ThermoFisher Scientific) in a total volume of 20 μl per reaction. The cDNA product was diluted 10‐fold in water and used at this concentration for qPCR. Qualitative PCR was performed using SsoAdvanced Universal SYBR Green qPCR Master Mix (Bio‐Rad), with primers at a final concentration of 500 n*M* from a 10 μ*M* stock. Diluted cDNA (2.5 μl) was used per reaction, and all reactions were performed in triplicate in a total volume of 10 μl. Primer sequences are shown in Supplementary Table 1, available on the *Arthritis & Rheumatology* web site at http://onlinelibrary.wiley.com/doi/10.1002/art.40659/abstract. A 2‐step qPCR cycle with a Bio‐Rad iCycler qPCR machine was used for target amplification, according to the instructions of the manufacturer for SSoAdvanced Universal SYBR Green Master Mix, and CFX Manager was used for analysis.

## Results

#### Reduced regenerative potential shown by SGSCs from patients with primary SS

**Figure 1:**
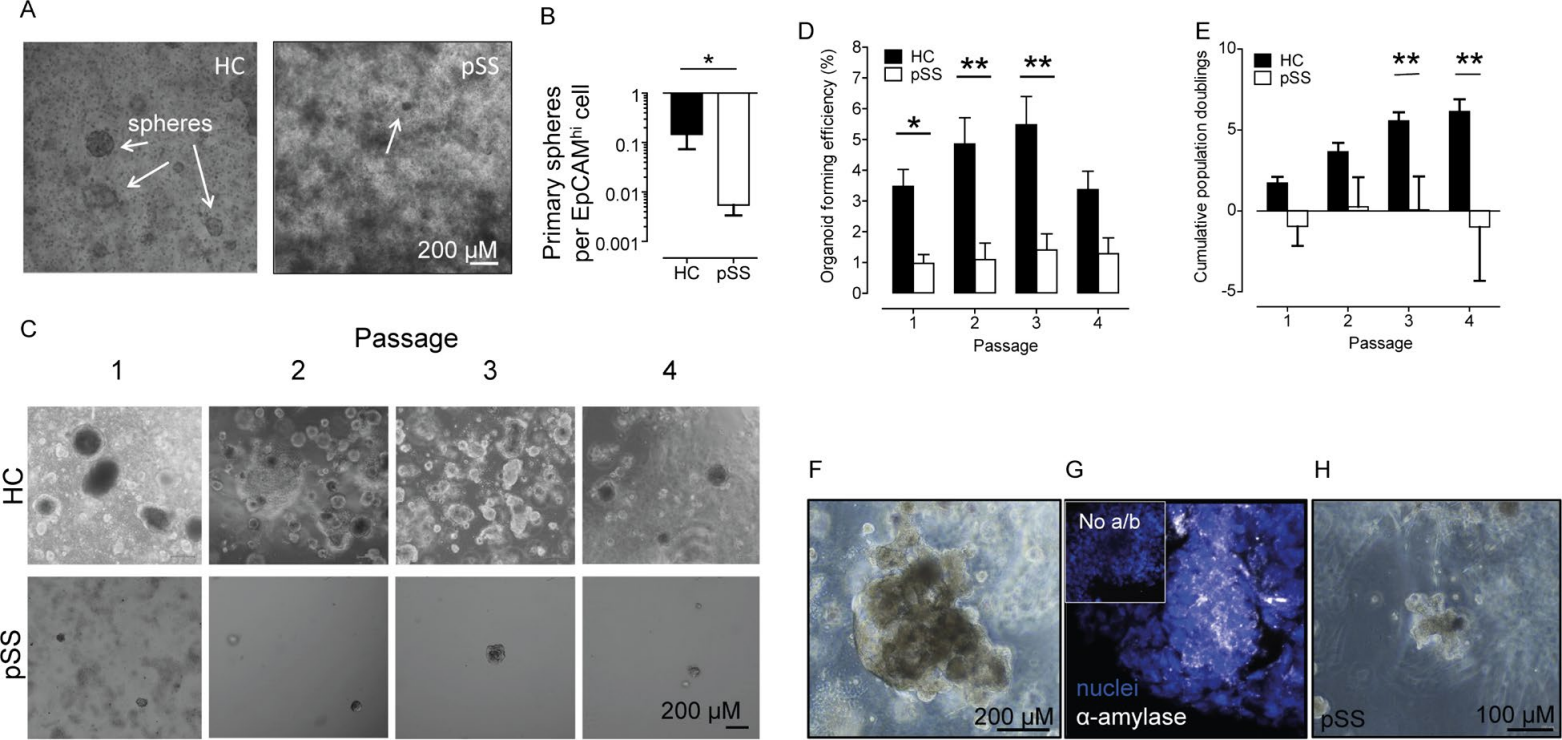
Salivary gland stem cells (SGSCs) from patients with primary Sjögren's syndrome (pSS) show reduced regenerative potential. **A**, Microscopy of primary spheres isolated from SGSCs from a healthy control (HC) (left) and a patient with primary SS (right). **Arrows** show organoids. **B**, Quantification of primary sphere yield per epithelial cell adhesion molecule (EpCAM ^high^) cell in SGSC isolates from biopsy samples from healthy controls (n = 6) and patients with primary SS (n = 9). * = *P* < 0.05 by Student's *t*‐test. **C**, Microscopy of organoid cultures of SGSCs from a healthy control and a patient with primary SS. **D**, Organoid‐forming efficiency of SGSCs in cultures of biopsy tissue from healthy controls (n = 27 at passages 1–4) and patients with primary SS (n = 12, 16, 9, and 6 at passages 1–4, respectively). * = *P* < 0.05; ** = *P* < 0.01 by two‐way analysis of variance (ANOVA) with Bonferroni post hoc testing. **E**, Cumulative population doublings of SGSCs from healthy controls (n = 26 at passages 1–3; n = 24 at passage 4) and patients with primary SS (n = 10, 5, 4, and 2 at passages 1–4, respectively). ** = *P* < 0.01 by two‐way ANOVA with Bonferroni post hoc testing. **F**, Phase‐contrast microscopy of mature organoids formed from SGSCs from a healthy control. **G**, Immunocytochemical staining of acinar cell–associated amylase in a healthy control SG sample–derived mature organoid. **Inset** shows a healthy control SG sample without anti‐amylase antibody (a/b) to demonstrate staining specificity. **H**, Diminished ability to form mature organoids from primary SS SGSCs. Values in **B**,** D**, and **E**, are the mean ± SEM.

We began by isolating SGSCs from parotid SG biopsy samples from control patients with healthy SGs and patients with primary SS fulfilling the ACR/EULAR classification criteria 20. SGSCs were initially cultured from processed biopsy samples as primary spheres in Wnt‐containing medium. Three to 5 days later, spheres were dispersed to single SGSCs and expanded in a “self‐renewal assay.” The cell suspension generated by the isolation process from primary SS biopsy samples contained significantly fewer epithelial cells than healthy control biopsy samples and significantly more CD45+ leukocytes, based on cell morphology and immunostaining on cytospots (Supplementary Figures 2 and 3A–C, available on the *Arthritis & Rheumatology* web site at http://onlinelibrary.wiley.com/doi/10.1002/art.40659/abstract). As previously reported for minor SGs (23), we detected a high proportion of B cells and a predominance of CD4+ T cells within the flow cytometry–defined CD45+ fraction of the biopsy isolate (Supplementary Figure 3D and E, available at http://onlinelibrary.wiley.com/doi/10.1002/art.40659/abstract). SGSCs are EpCAM^high^ in nature. The number of both spheres generated per EpCAM^high^ cell and yield of spheres per milligram of biopsy sample was significantly lower (a 10‐fold difference) in samples from patients with primary SS compared to the healthy samples (Figures 1A and B, and Supplementary Figure 3F, available at http://onlinelibrary.wiley.com/doi/10.1002/art.40659/abstract).

(Data are presented as normalized to milligrams of tissue to take into account the larger size of the healthy SG biopsy samples obtained.) Primary sphere yield was not correlated with focus score (lymphocytic infiltration) (Supplementary Figure 3G, available at http://onlinelibrary.wiley.com/doi/10.1002/art.40659/abstract), supporting the notion that infiltration does not determine SG function. We have previously demonstrated that SGSC yield decreases with age and that more SGSCs are present closest to the facial nerve in the parotid gland 21, 24. Neither donor age nor biopsy site was responsible for the decreased yield of SGSCs from primary SS biopsy samples (Supplementary Figures 3H and I, available at http://onlinelibrary.wiley.com/doi/10.1002/art.40659/abstract). Stem cells are classically defined by their ability to proliferate and differentiate. When SGSCs from primary SS biopsies were cultured as organoids to assess their proliferation capacity, we observed a significantly (up to 5 times) lower self‐renewal capability compared to healthy samples (Figures 1C–E) Thus,. FACS selection of EpCAM^high^ cells from primary SS biopsies, after removal of infiltrating leukocytes, did not rescue the self‐renewal potential of primary SS SGSCs (Supplementary Figure 3, http://onlinelibrary.wiley.com/doi/10.1002/art.40659/abstract), indicating that the sole presence of CD45+ cells in SGs of patients with primary SS is not responsible for the regenerative deficits observed.

Healthy SGSCs could be induced to proliferate and differentiate from organoids into α‐amylase–expressing mature organoids (Figures 1F and G). The lack of proliferative capabilities of SGSCs from primary SS biopsies was reflected also in their greatly diminished ability to form mature organoids (Figure 1H). These data imply that the relatively few SGSCs present in primary SS SGs also harbor defects in differentiation ability.

#### Extensive replicative history of SGSCs in primary SS

**Figure 2:**
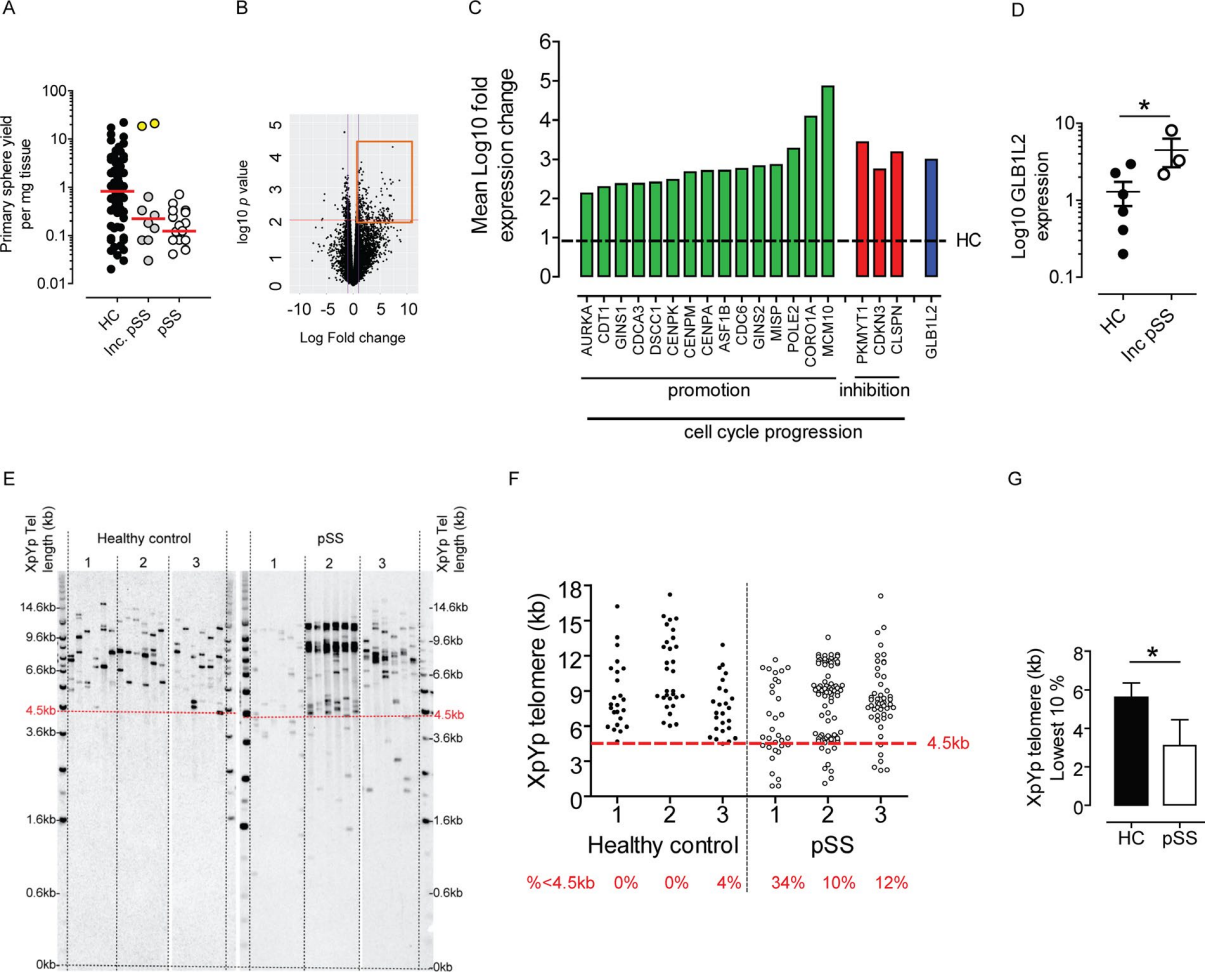
Salivary gland stem cells (SGSCs) from patients with primary Sjögren's syndrome (pSS) are more likely to be senescent. **A**, Primary sphere yield in SGSCs from healthy controls (HC) (n = 73), patients with incomplete (Inc.) primary SS (n = 10), and patients with primary SS (n = 18). Symbols represent individual spheres. Yellow circles show SGSCs with unusually high yield. Red lines show the median. The primary SS group from Figure 1B is used for comparison. **B**, Volcano plot resulting from RNA sequencing analysis comparing SGSC transcriptomes in biopsy samples from healthy controls and patients with incomplete primary SS. Boxed area denotes genes whose expression is ≥log^10^ 2‐fold higher in primary SS SGSCs. Red line shows *P* value cutoff threshold (*P* < 0.01). **C**, Up‐regulation of cell cycle progression promotion genes (green) and inhibition genes (red) identified from RNA sequencing, including the β‐galactosidase–like gene (GLB1L2) (blue). Broken line represents the mean expression level in healthy controls (n = 6 individual samples from healthy controls; n = 3 samples from patients with primary SS. **D**, Raw expression values for GLB1L2. Symbols represent individual samples from healthy controls (n = 6) and patients with primary SS) (n = 3). Bars show the mean ± SEM. **E**, Single telomere length analysis of SGSC telomere lengths in biopsy samples from healthy controls and patients with biopsy‐proven primary SS, showing outlying small (<4.5 kb) telomeres (red dotted line) in primary SS SGSCs. **F**, Quantification of telomere lengths in SGSCs from healthy controls and patients with primary SS. Red text denotes percentage of telomeres with length <4.5 kb (broken line). Symbols represent individual samples. **G**, Length analysis of lowest 10% of telomeres in SGSCs from healthy controls and patients with biopsy‐proven primary SS (n = 3 per group). Values are the mean ± SEM. * = *P* ≤ 0.05 by Student's *t*‐test.

In order to elucidate the early events in SG pathology development in primary SS that are not influenced by mass lymphocytic infiltration, we focused on patients classified as having incomplete primary SS. These patients have some hallmarks of primary SS (outlined in Supplementary Table 2, available on the *Arthritis & Rheumatology* web site at http://onlinelibrary.wiley.com/doi/10.1002/art.40659/abstract) but do not have a positive lymphocyte focus score. Also, the patients with incomplete primary SS were not taking medication known to cause dry mouth symptoms, they all had recorded symptoms of dry eyes and mouth associated with primary SS development, and they did not fulfill the ACR/EULAR criteria. We consider these features indicative of early SG pathology development in primary SS. When SGSCs were isolated from the patients with incomplete primary SS we observed that the primary sphere yield from a small proportion (2 of 10) of these biopsy samples was markedly greater than the median of the healthy control samples (Figure 2A). The yield of primary spheres from the remaining biopsy samples was comparable to the biopsy yield from the patients with primary SS. Reasoning that the 2 (of 10) patients with the high yield represent an earlier disease stage (25), we theorized that SGSCs receive mitotic stimuli early in primary SS. We performed RNA sequencing on organoids cultured from patients with incomplete primary SS to investigate early events in primary SS, and we observed a cohort of 101 significantly up‐regulated genes in SGSCs from biopsy‐negative patients compared to healthy control samples (*P* < 0.01, log_10_‐fold change ≥2) (Figure 2B).

On further examination, we found that 18 of these genes were involved in cell cycle progression (both its promotion and inhibition) and DNA replication (Figure 2C and Supplementary Figure 4, available on the *Arthritis & Rheumatology* web site at http://onlinelibrary.wiley.com/doi/10.1002/art.40659/abstract). As shown in Figures 2C and D, the β‐galactosidase–like gene GLB1L2 was also significantly up‐regulated. Beta galactosidase expression is associated with cellular senescence and aging. Hypothesizing that SGSCs in primary SS disease progression become senescent, we examined the telomere lengths of organoids cultured from patients with primary SS with positive SG biopsy evaluations (i.e., with lymphocytic infiltration), representing a later phase of primary SS in terms of SG pathology. STELA analysis of telomere length revealed short telomeres of <4.5 kb in length in SGSCs from biopsy‐positive patients with primary SS (Figures 2E and F(and clinical characteristics shown in Supplementary Table 2, available on the *Arthritis & Rheumatology* web site at http://onlinelibrary.wiley.com/doi/10.1002/art.40659/abstract). The mean length of the lowest 10% of telomeres in the SGSCs of healthy control samples was significantly greater (5.58 kb) compared to primary SS SGSCs (3.10 kb) (Figure 2G), suggesting that primary SS SGSCs have a more extensive replicative history. The mean ages of healthy control sample donors and primary SS SGSC donors in whom telomere analysis was performed were 77.3 and 61.5 years, respectively, confirming that telomere difference was not due to the advanced age of SGSC donors with primary SS.

#### Proinflammatory cytokines include proliferation and differentiation of healthy SGSCs

**Figure 3:**
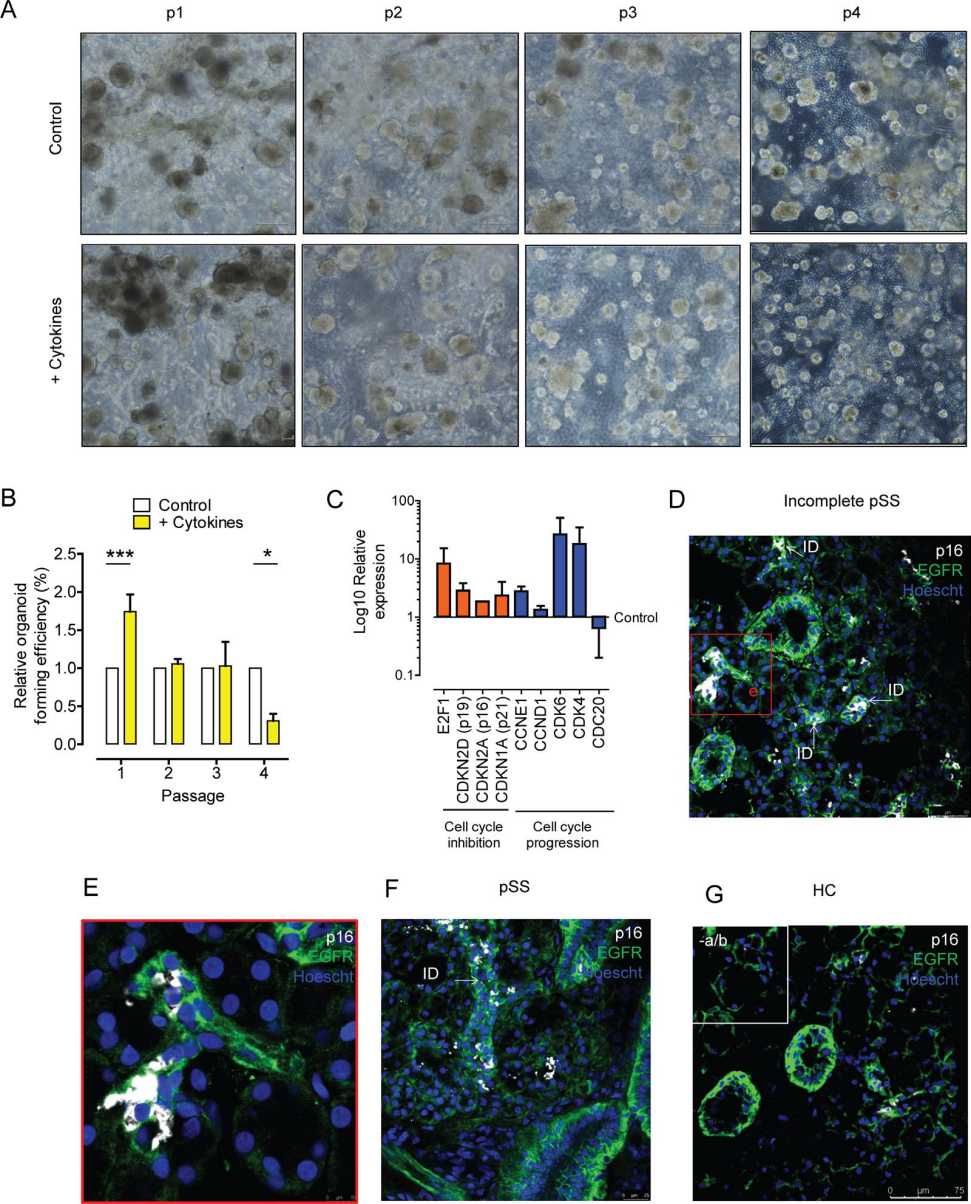
Parotid salivary gland stem cell (SGSC) organoid cultures proliferate upon exposure to a proinflammatory cytokine cocktail and express cell cycle and senescence genes. P16+ senescent cells localize to the intercalated ducts (IDs) in SG tissue from patients with incomplete primary Sjögren's syndrome (pSS) and patients with primary SS. **A**, Phase‐contrast microscopy of SGSCs from a healthy control at passages 1–4, incubated with (+ Cytokines) and without (Control) the proinflammatory cytokine cocktail. **B**, Quantification of organoid formation efficiency of SGSCs exposed to proinflammatory cytokines compared to control cells; (n = ≥7 separate cell sample isolations at each passage). Bars show the mean ± SEM. * = *P* < 0.05; *** = *P* < 0.001 by two‐way analysis of variance. **C**, Expression of cell cycle–associated genes in SGSCs exposed to proinflammatory cytokines. Cells were harvested at the end of passage 1 for quantitative polymerase chain reaction analysis. Bars show the mean ± SEM (n = 2 separate cell sample isolations). **D–G**, Immunohistochemical staining for p16 senescence marker in SG tissue from a patient with incomplete primary SS (**D**;** D inset** shown in **E**), a patient with primary SS (**F**), and a healthy control (HC) (**G**), counterstained with epithelial growth factor receptor (EGFR) to mark all ductal cells. **Inset** in **G** shows SG tissue from a healthy control without antibody (−a/b) staining, to demonstrate specificity. Ages of tissue donors were 50, 73, and 31 years for the healthy control, patient with incomplete primary SS, and patient with primary SS, respectively, indicating that the increase in p16+ ID cells could not be attributed to the advanced age of the donor.

Primary SS is an autoimmune disease associated with the glandular presence of classic proinflammatory cytokines, as exemplified by IFNα, TNF, and IL‐6 23. Proinflammatory cytokines within the glandular tissue could provide mitotic signals, driving SGSC exhaustion in primary SS and leading to a senescent, aging‐like phenotype and ultimately hyposalivation. Considering the low yield of SGSCs from patients with primary SS, and in order to model the earliest phases of primary SS, we employed healthy control SGSC cultures to investigate this hypothesis. Quantitative PCR and immunostaining of healthy control SGSC organoids at passage 2 demonstrated that healthy control SGSCs express receptors for the proinflammatory cytokines IFNα, TNF, and IL‐6 (see Supplementary Figure 5 and [for primers] Supplementary Table 1, available on the *Arthritis & Rheumatology* web site at http://onlinelibrary.wiley.com/doi/10.1002/art.40659/abstract). When healthy control SGSCs from passages 1–4 were incubated with a cocktail of proinflammatory cytokines at concentrations matching those found in the serum of patients with primary SS (IFNα 500 pg/ml, TNF 40 pg/ml, and IL‐6 30 pg/ml) 26, we observed initially a significant increase in organoid formation efficiency, followed by a decrease to significantly below the levels in control cells (Figures 3A and B). Incubation with single cytokines did not induce significant proliferative effects, even at higher doses (see Supplementary Figure 6, available at http://onlinelibrary.wiley.com/doi/10.1002/art.40659/abstract).

At passage 1 following cytokine exposure, expression of genes promoting cell cycle progression (CDK4, CDK6, and CDC20), inhibiting cell cycle (E2F1 and CDKN2D), and promoting senescence (p16 and p21) was up‐regulated (Figure 3C). Through definition of SGSC subsets using cell surface markers and costaining with the proliferation marker Ki‐67 (see Supplementary Figures 7A and B, available at http://onlinelibrary.wiley.com/doi/10.1002/art.40659/abstract), we also showed that SGSCs resident in the basal layer of striated ducts (BSD cells) were responsible for the proliferation observed (Supplementary Figure 7C, available at http://onlinelibrary.wiley.com/doi/10.1002/art.40659/abstract). We also suggest that proinflammatory cytokines induce differentiation of BSD cells into intercalated ducts (ID cells) (Supplementary Figure s 7C and D. Finally, p16 immunostaining was performed on sections of SG tissue in order to determine where senescent cells were located in situ; p16+ cells were found mostly in intercalated ducts in incomplete and complete primary SS tissue. In contrast, p16+ cells in healthy SGs were found dispersed through the tissue (Figures 3D–G), illustrating their full differentiation potential.

## Discussion

The origins of hyposalivation development in primary SS have never been fully elucidated, although many studies have now firmly established that its development cannot be fully explained by the extent of lymphocytic infiltration 3, 4, 5, 6, 7, 8, 9. Using SGSCs as a tool to probe SG dysfunction in primary SS, we showed in the present study that parotid gland biopsy samples from patients with primary SS contain fewer SGSCs with reduced proliferation, differentiation potential, and shortened telomeres. Shortened telomeres imply that the SGSC pool has an extensive replicative history, the reason for which we propose is 2‐fold.

First, the parenchymal epithelium, e.g., the non‐stem ductal cells and saliva‐producing acinar cells in primary SS, have been demonstrated to undergo enhanced levels of apoptosis, from sources intrinsic and extrinsic to the cells themselves. Extrinsically, the action of cytokines, cytotoxic T cells, and natural killer cells all promote apoptosis 27. Additionally, a disorganized extracellular matrix in primary SS SGs may account for acinar cell loss by anoikis 28. Epithelial cells have been shown to intrinsically express defective levels of the antiinflammatory mediator peroxisome proliferator–activated receptor γ, resulting in increased activity of the NF‐κB and IL‐6 pathways, but also rendering them more susceptible to cell death 29, 30, 31, 32, 33, 34. Similarly, levels of the ubiquitin‐editing protein A20, a negative regulator of NF‐κB, were down‐regulated in SG epithelial cells from patients with primary SS compared to healthy subjects 35. Therefore, depletion of the parenchymal cell pool via intrinsic and extrinsic mechanisms together likely stimulates SGSC proliferation and differentiation into acinar cells, in an attempt to maintain the saliva‐producing capacity of the SGs.

Second, as we have demonstrated here, proinflammatory cytokines exert a direct effect on proliferation of SGSCs. In other model systems, and most extensively in the well‐characterized intestinal stem cell niche, proinflammatory cytokines have also been shown to exert a proliferative effect, mediated by modulation of the stem cell–associated Wnt, Notch, and Yes‐associated protein/transcriptional co‐activator with PDZ‐binding motif (YAP/TAZ) pathways, suggesting that cross‐talk between stem cells and the elements of the immune system may underlie many disease manifestations 36, 37, 38, 39, 40. Cytokine production in the case of primary SS may be derived from neighboring epithelial cells signaling in a paracrine manner. The production and secretion of proinflammatory cytokines by epithelial cells has been demonstrated in long‐term epithelial culture systems and in situ 32, 41, 42, 43, 44. Following the release of damage‐ and pathogen‐associated molecular patterns, e.g., molecules such as high mobility group box chromosomal protein 1 and viral antigens, pattern recognition receptors on epithelial cells may be activated, culminating in epithelial cell autonomous NF‐κB pathway activity, cytokine production, and paracrine signaling to neighboring SGSCs 33, 34. Indeed, the dysregulated NF‐κB pathway seen in primary SS may account for the sustained proinflammatory cytokine production by glandular epithelial cells 33, 35.

One prevailing theory regarding stem cells dictates that under healthy conditions, SGSCs reside in the striated ducts, proliferate and differentiate into intercalated ducts, and then finally into saliva‐producing acinar cells. We have demonstrated the presence of senescent cells in intercalated ducts of primary SS SGs. This presence suggests a blockade in the ability of SGSCs to further differentiate into acinar cells, presumably due to having reached their regenerative limit, similar to the poor mature organoid differentiation potential we demonstrated in vitro. Clinically, our data suggest that screening patient SGs or saliva for senescence biomarker expression may indicate the extent of SGSC exhaustion. We further suggest that clinical interventions aimed at preventing hyposalivation development need to occur before the appearance of high levels of senescent markers in SGs or saliva. The present study findings also suggest, critically, that effective interventions to cure established hyposalivation by targeting the inflammatory process are not likely to involve only immune signal blockade, but rather the replenishment of SGSC stocks in conjunction with resolving the inflammation. Plausible strategies include the use of induced pluripotent stem cell technologies in the manufacture of SGSCs.

In conclusion, we have demonstrated for the first time an aging phenotype as a potential causative agent for the lack of SG repair in the autoimmune disease primary SS, and link this finding to possible future clinical strategies.

## Author Contributions

All authors were involved in drafting the article or revising it critically for important intellectual content, and all authors approved the final version to be submitted for publication. Dr. Pringle had full access to all of the data in the study and takes responsibility for the integrity of the data and the accuracy of the data analysis.

### Study conception and design.

Pringle, Wang, Verstappen, Terpstra, Zhang, He, Patel, Jones, Baird, Spijkervet, Vissink, Bootsma, Coppes, Kroese.

### Acquisition of data.

Pringle, Wang, Verstappen, Terpstra, Zhang, He, Patel, Jones.

### Analysis and interpretation of data.

Pringle.

## Role of the Study Sponsor

Bristol‐Myers Squibb had no role in the study design, the data interpretation, the writing of the manuscript, or the decision to submit the manuscript for publication. Bristol‐Myers Squibb did perform RNASeq data analysis. Publication of this article was not contingent upon approval by Bristol‐Myers Squibb.
