# Supplementary material for: Salivary Gland Stem Cells Age Prematurely in Primary Sjögren's Syndrome
Source: Arthritis Rheumatol. 2018 Dec 27;71(1):133–42. doi: 10.1002/art.40659 (PMC6607019; doi:10.1002/art.40659)
Supplement: Supplementary file 1 [file ART-71-133-s001.docx]

Salivary Gland Stem Cells Age Prematurely in Primary Sjögren’s syndrome

Supplementary Figures.

Sarah Pringle^1^*, Xiaoyan. Wang^1,^, Gwenny. M.P.J. Verstappen^1^, Janneke. H. Terpstra^1^, Clarence. K. Zhang^2^, Aiqing. He^3^, Vishal. Patel^3^, Rhiannon. E. Jones^4^, Duncan. M. Baird^4^, Fred. K. L. Spijkervet^5^, Arjan. Vissink^5^, Hendrika. Bootsma^1^, Robert. P. Coppes^67†^ and Frans. G.M. Kroese. ^1†^

Supplementary Figures:

**
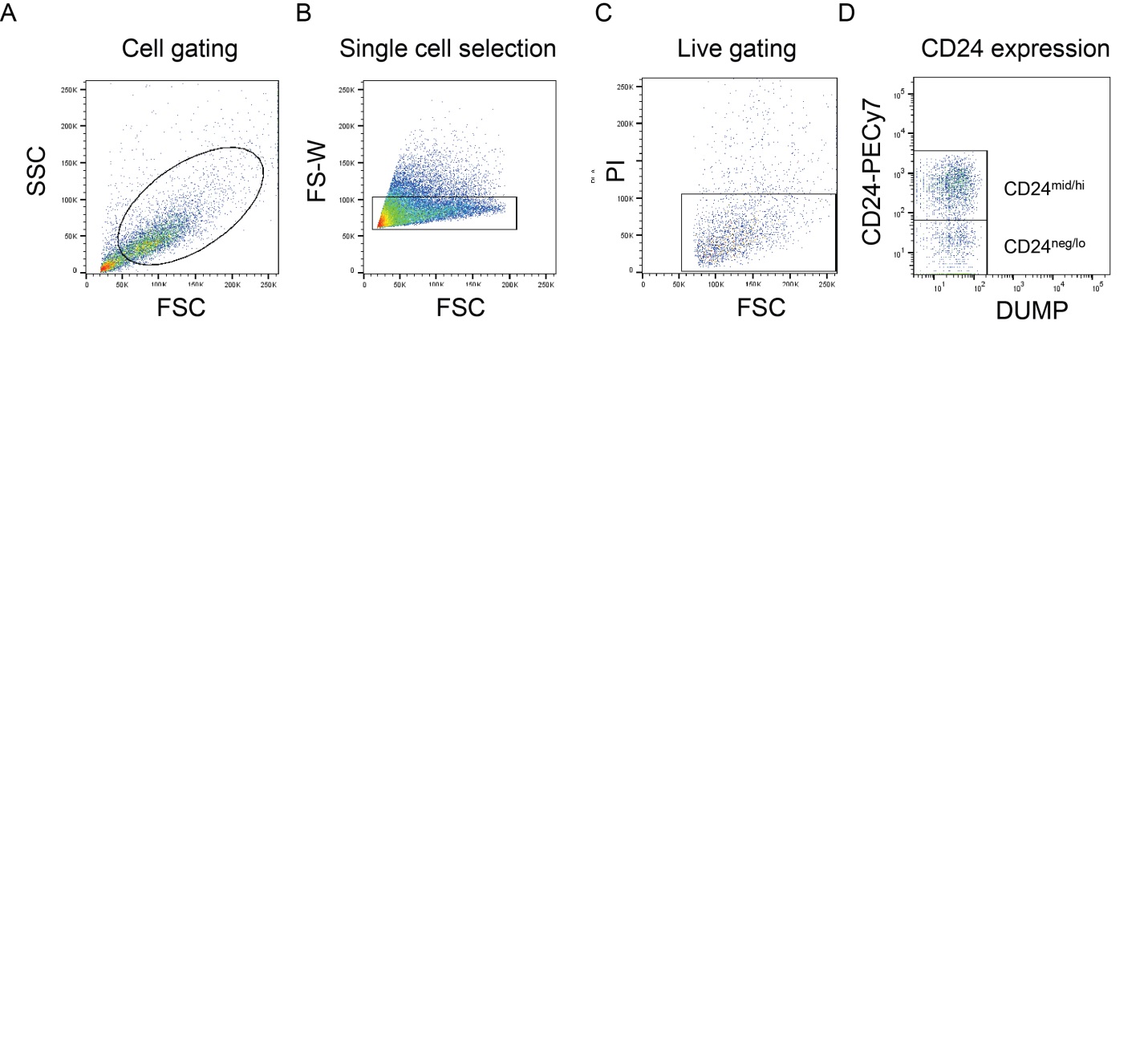
**

**Fig. S1**. Gating strategy used for flow cytometry. Cells were **A**) first gated on forward and side scatter axes, **B**) doublets were discriminated, **C**) live dead discrimination performed with Fixable Viability Dye or Propidium Iodide and gated for analysis of CD24 or Ki67 expression.


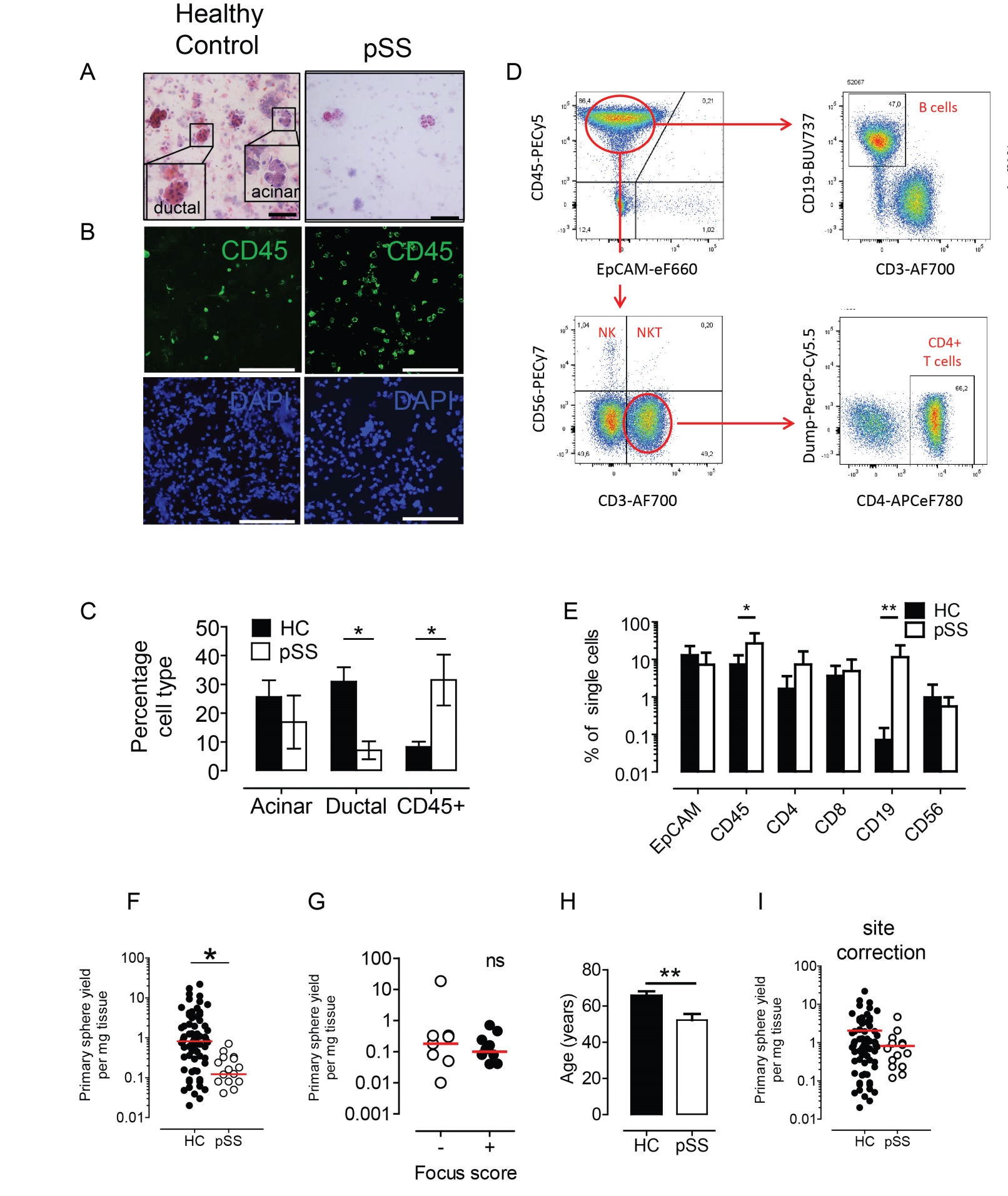


**Fig. S2.** SGSC isolate from pSS biopsies contains less ductal cells, more CD45+ leukocytes and generates less organoids per EpCAM+ cell. **A**) Haematoxylin and eosin staining of cytospots made from final cell solution after SGSC isolation protocol. Ductal and acinar cells were identified on basis of morphology, and indicated in inset boxes in healthy control panel. **B**) Proportion of CD45+ leukocytes was determined by immune labeling of cytospots. Top row of B shows single CD45 staining, bottom row all cells, identified by DAPI nuclear staining. **C**) Quantification of proportion of acinar, ductal and CD45^+^ cells. *n* = 10 for healthy controls, 5 for ACR-EULAR criteria diagnosed primary Sjogren’s syndrome samples. Error bars are S.E.M. Scale bars in all images are 100 µM. **D**) Flow cytometry analysis of cell isolate from pSS biopsies, showing high proportion of CD45^+^ cells, B cells (CD19^+^) and CD4^+^ T Cells (CD3^+^CD4^+^). **E**) Summary of proportion of EpCAM^+^ cells, CD45^+^ leukocytes, CD4^+^ T cells, CD8^+^ T cells, CD19^+^ B cells, and CD56^+^ NK cells in isolate generated from processing of pSS and healthy control salivary gland biopsies. *n* = minimum of 6 patients per group per marker. * = *p* < 0.05 ** *p* < 0.01, student’s *t*- test. **F**) Primary sphere yield per mg in pSS biopsies compared to HCs. Line represents median value. *n*=29 for HC; 18 for pSS. *= *p* value of 0.0279, student’s *t*-test **G**) Primary sphere yield from ACR-EULAR classified pSS biopsies does not differ with positive or negative focus score. *n* = 9 and 10 for focus score negative and positive pSS biopsies respectively. **H**) Primary sphere yield from HC and pSS biopsies corrected for age of patient. *n* = 5 and 17 for HC and pSS donors respectively. HC donors are significantly older that pSS donors, suggesting that lower yield from pSS biopsies is not due to advanced age of pSS donors compared to HCs. **I**) Primary sphere from HC and pSS biopsies corrected for optimal site of biopsy harvest. *n* = 72 for HC group and 17 for pSS group.

**
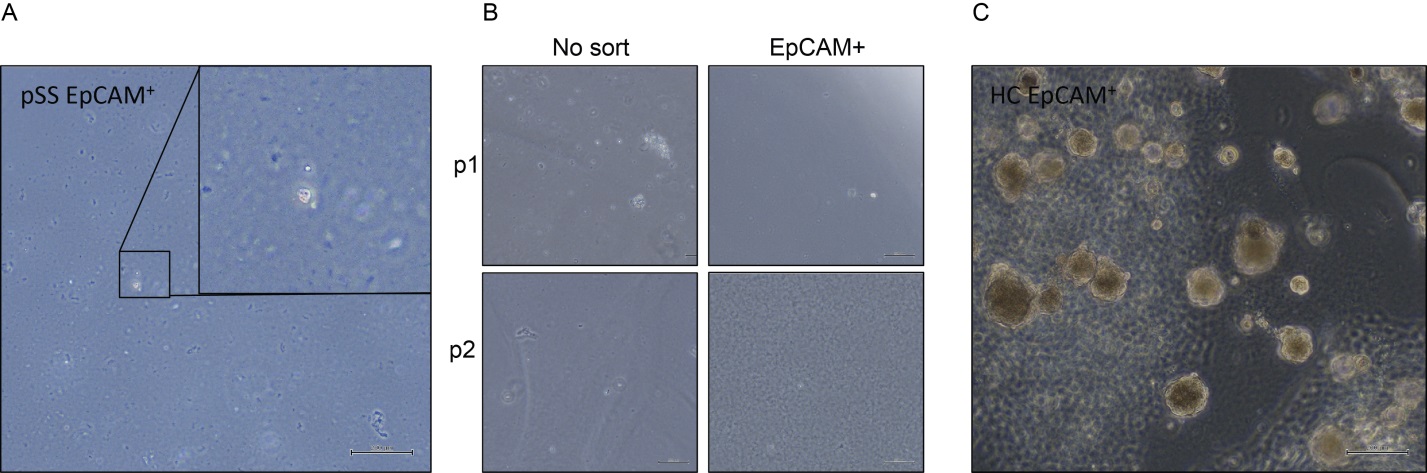
**

**Fig. S3.** Removal of CD45^+^ infiltrate from pSS isolate does not rescue proliferative potential of pSS-SGSCs. **A**) EpCAM^+^ cells isolated from a pSS biopsy and cultured. Presence of phase-bright (alive) cells can be observed, which do not appear to proliferate. **B**) Attempts at passaging EpCAM+ cells sorted from pSS biopsies did not rescue growth. **C**) EpCAM^+^ cells sorted and passaged from healthy control (HC) biopsies grow as organoids following FACS sorting. Lack of growth of pSS derived EpCAM^+^ cells is therefore not due to damage during sorting.


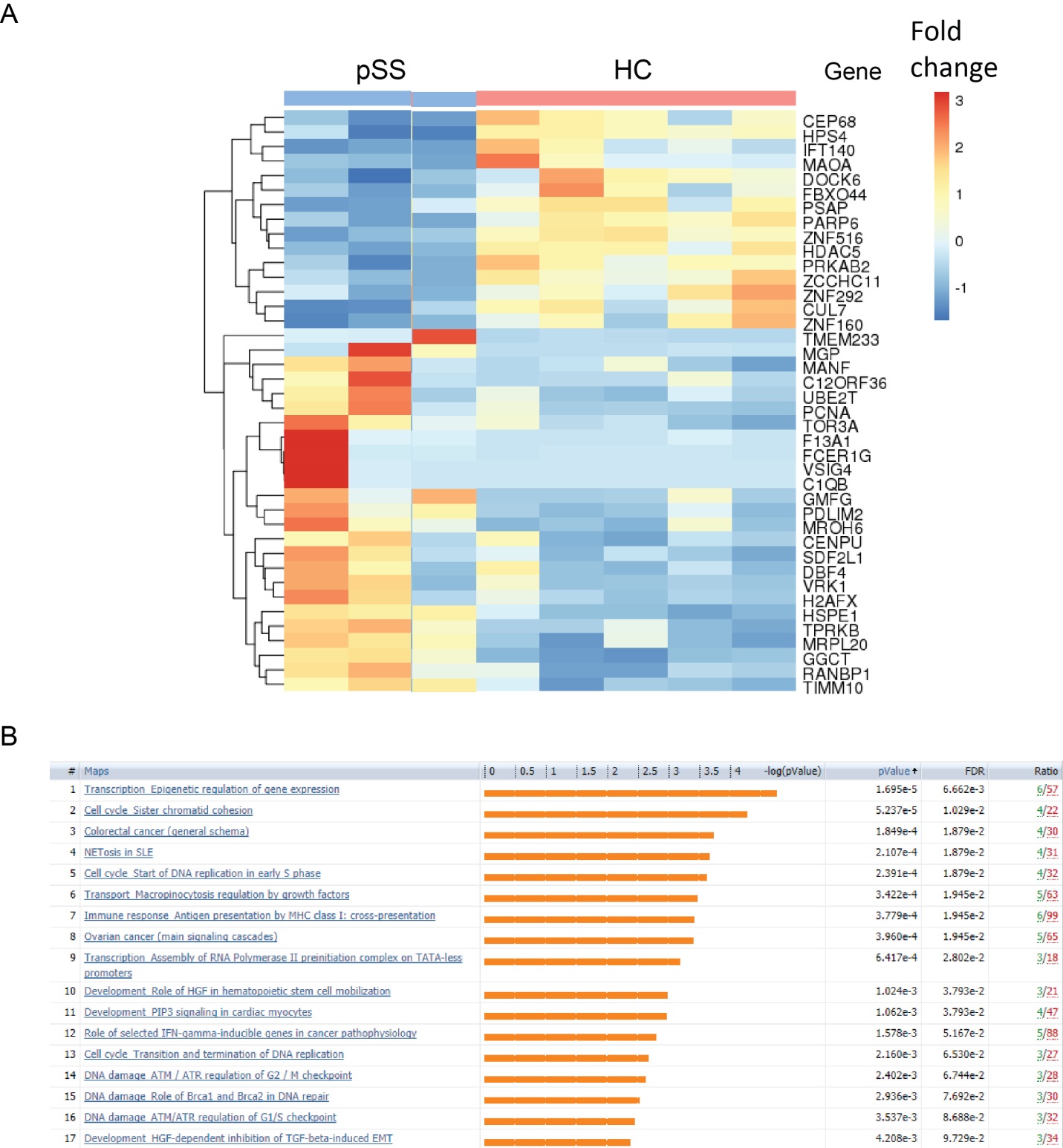


**Fig. S4.** Further RNASeq profiling of SGSCs reveals upregulation of pathways involved in cell cycle regulation and DNA damage*.* **A**) Heatmap of 40 differentially expressed genes (*p* < 0.001) between pSS and HCs. Colour coding of differential expression fold changes is given in the legend. **B**) Metacore pathway analysis of RNASeq data identified potential pathways enriched in SGSCs from pSS SG biopsies. (p < 0.005, based on 222 genes).

**
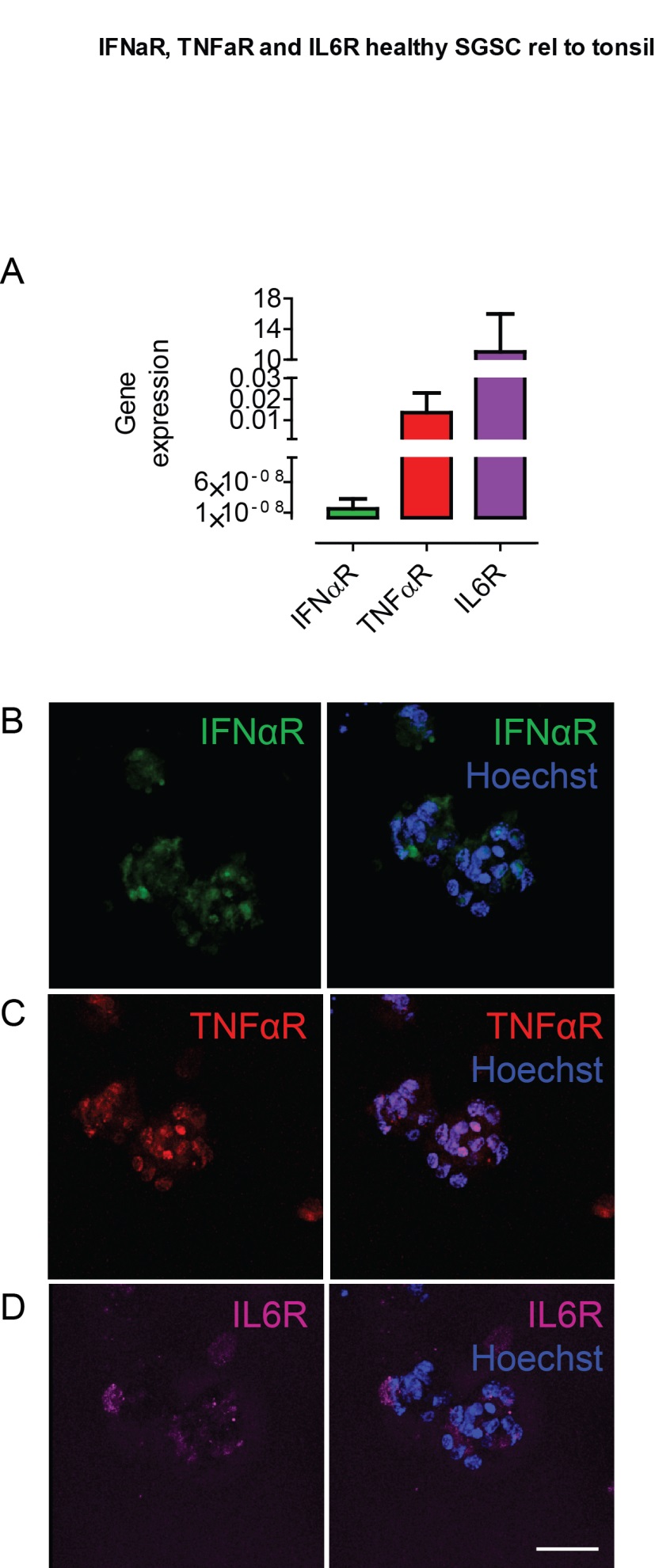
**

**Fig. S5.** SGSCs express IFNα, TNFα and IL6 receptors*.* **A**) Expression of IFNα, TNFα and IL6 receptors as determined by qPCR. *n* = 3 separate patient biopsies. Inflamed human tonsil cDNA was used as a positive control, and all expression expressed in relation to tonsil. **B-D**) Whole mount microscopy of healthy SGSCs showing expression of the IFNα, TNFα and IL6 receptors. Receptor colour as indicated in panels. First panel shows single colour channel used for the relevant receptor, second a merged image with Hoechst. Scale bar = 50 µM.

**
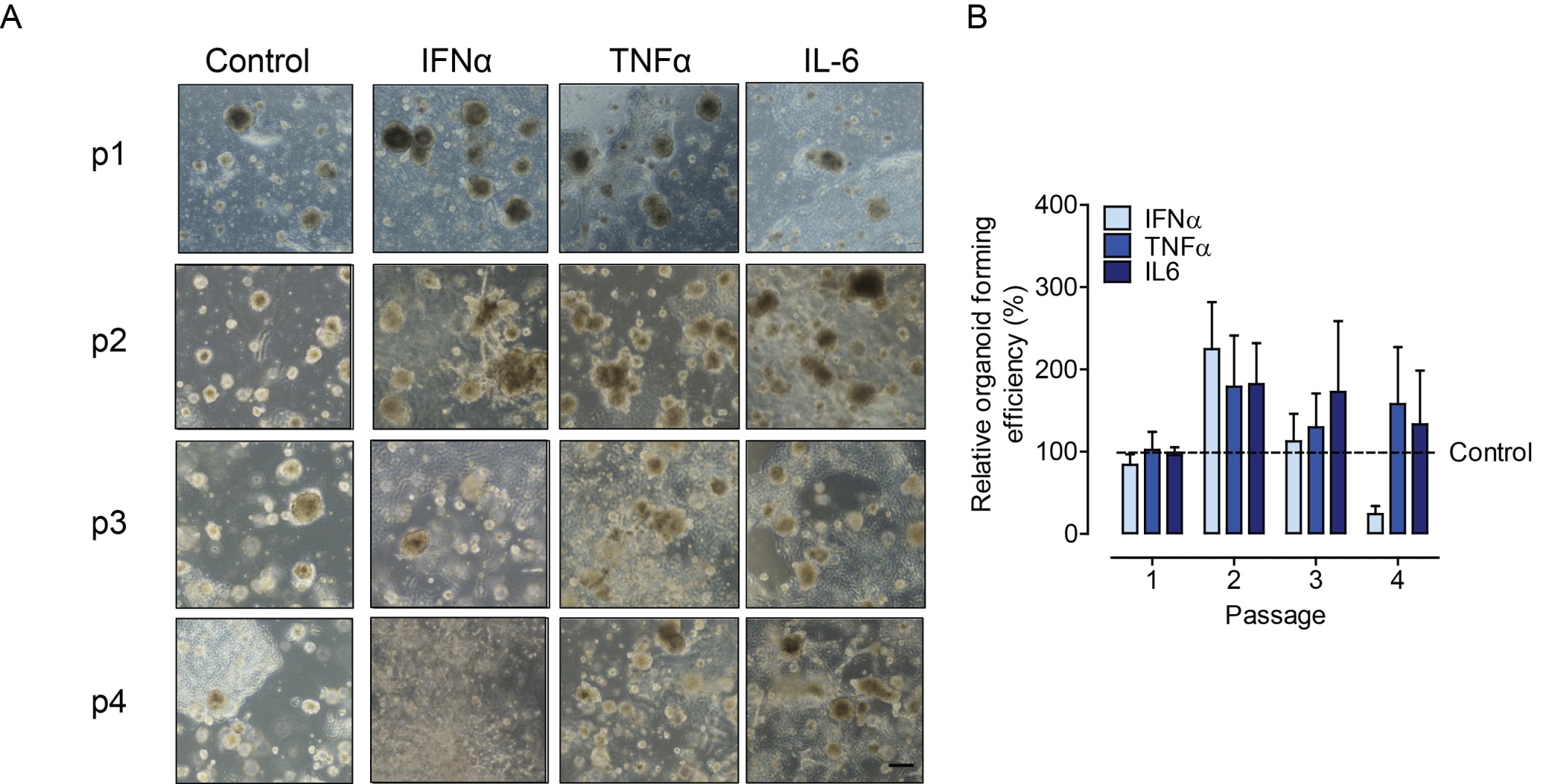
**

**Fig. S6.** Incubation of SGSCs with single proinflammatory cytokines IFNα, TNFα and IL6 does not induce significant proliferation*.* **A**) Representative phase contrast microscopy of healthy control SGSCs organoids exposed to IFNα (1 ng/mL), IFNɣ (0.1 pg/mL), TNFα (1 ng/mL) and IL-6 (10 ng/mL) at passages 1-4. Scale bar represents 200 µM and applies to all images. **B**) Quantification of proliferation index of cytokine co-cultures. A minimum of 4 distinct biopsies were incubated with each cytokine ,and proliferation index determined at every passage. Bar height represents mean, error bars represent SEM. Two Way-ANOVA testing was performed, * = p < 0.05.

**
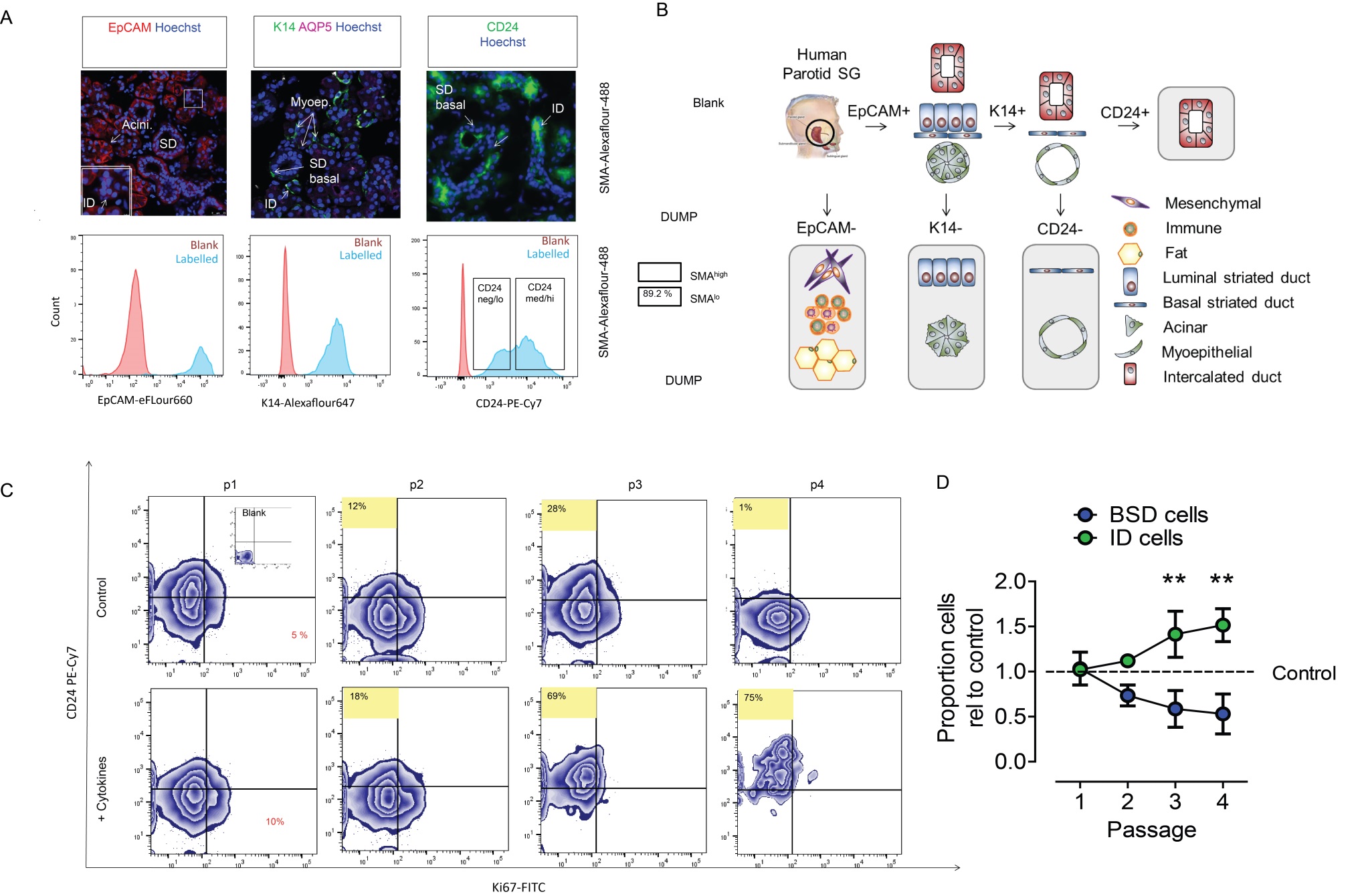
**

**Fig. S7.** SGSC cultures contain a mixture of basal striated duct cells and intercalated duct cells. Proinflammatory cytokine exposure induces proliferation of the basal striated duct cells, and their differentiation into intercalated duct cells. **A-C**) Immunostaining of healthy parotid tissue for EpCAM, K14 and CD24, respectively. K14 labelling is counterstained with AQP5 for definition of tissue. ID = intercalated duct. SD = striated duct. **D-F**) Flow cytometric analysis of SGSCs culture for expression of EpCAM, K14 and CD24 respectively. **G,H**) Flow cytometric analysis of a SGSC culture for expression of smooth muscle actin (SMA), a marker protein of myoepithelial cells. SGSC culture were mid-positive to SMA, as opposed to established high positivity of myoepithelial cells. **I**) Schematic showing logical basis for statement that SGSC organoid cultures contains mixture of SGSC from the striated and intercalated ducts, which can be defined ultimately by CD24 expression. Stock human head image adapted from the Blausen library. **J**) Flow cytometric analysis of Ki67 and CD24 expression in SGSCs exposed to proinflammatory cytokines, compared to untreated controls. An initial increase in proliferation (Ki67^+^) of basal striated duct (BSD) cells was observed. Red text highlights increased proportion of Ki67^+^ ID cells at p1 following cytokine exposure. Yellow boxed text highlights increasing proportion of CD24^+^ ID cells following cytokine exposure. Inset box shows blank staining control. **K**) Quantification of proportion of BSD cells and ID cells following SGSC treatment with cytokines. *n* = ≥ 4 separate patients isolations per group and time point. * p > 0.05, ** p > 0.01, Two Way ANOVA.

**Table S1**.

| Gene name | Gene symbol | Forward primer | Reverse primer |
| --- | --- | --- | --- |
| Tyrosine-3-monoxygenase | YWHAZ | ttcttgatccccaatgcttc | agttaagggccagacccagt |
| Interferon-α receptor 1 | IFNAR1 | cttcaggccaggagttcaag | ctgggtagggaaaggaaagc |
| Tumor necrosis factor-α receptor -1 | TNFRSR1A | ggagtgagaggccatagctg | gttcctttgtggcacttggt |
| Interluekin-6 receptor | IL6R | ggcaggttgtggaatctgtt | aagaccaccaactccacctg |
| Cyclin-dependent kinase 4 | CDK4 | gaaactctgaagccgaccag | aggcagagattcgcttgtgt |
| Cyclin-dependent kinase 6 | CDK6 | agagacaggagtggccttga | tgaaagcaagcaaacaggtg |
| Cyclin inhibitor 1A | CDKN1A | atgaaattcaccccctttcc | ccctaggctgtgctcacttc |
| Cyclin inhibitor 2A | CDKN2A | atatgccttcccccactacc | cccctgagcttccctagttc |
| Cyclin inhibitor 2D | CDKN2D | cttccaatccatctggcagt | ctcttgctggagagggtgac |
| E2F1 | E2F1 | atgttttcctgtgccctgag | atctgtggtgagggatgagg |
| Cyclin D1 | CCND1 | gaggaagaggaggaggagga | gagatggaagggggaaagag |
| Cyclin E1 | CCNE1 | agcggtaagaagcagagcag | tttgatgccatccacagaaa |
| Cell Division Cycle 20 | CDC20 | gtctgaccatgagcccagat | ctgaggtgatgggttggtct |

**Table S2.**

| Patient | Dry mouth complaints | Dry eye complaints | **Parotid**  **gland**  **histopathology** | SSA autoantibody titre | Unstimulated whole saliva drop | Ocular dryness | Echo score | ACR-EULAR score | Classification | Figure  used  in |
| --- | --- | --- | --- | --- | --- | --- | --- | --- | --- | --- |
|  |  |  |  |  |  |  |  |  |  |  |
| 1 | 1 | 1 | **0** | 1 | 0 | 0 | 0 | 3 | Incomplete pSS | 2a |
| 2 | 1 | 1 | **0** | 1 | 0 | 0 | 0 | 3 | Incomplete pSS | 2a |
| 3 | 1 | 1 | **0** | 0 | 1 | 1 | ND | 1 | Incomplete pSS | 2a |
| 4 | 1 | 1 | **0** | 0 | 1 | 1 | ND | 2 | Incomplete pSS | 2a |
| 5 | 1 | 1 | **0** | 0 | 1 | 1 | 0 | 2 | Incomplete pSS | 2a |
| 6 | 1 | 1 | **0** | 0 | 1 | 1 | 0 | 1 | Incomplete pSS | 2a |
| 7 | 1 | 1 | **0** | 0 | 1 | 1 | ND | 2 | Incomplete pSS | 2a |
| 8 | 1 | 1 | **0** | 0 | 1 | 1 | 0 | 2 | Incomplete pSS | 2a |
| 9 | 1 | 1 | **0** | 0 | 1 | 1 | 0 | 2 | Incomplete pSS | 2a |
| 10 | 1 | 1 | **0** | 1 | 1 | 1 | ND | 5 | pSS | 2a, b-d |
| 11 | 1 | 1 | **0** | 1 | 1 | 1 | 0 | 4 | pSS | 2b-d |
| 12 | 1 | 1 | **0** | 1 | 1 | 1 | 1 | 5 | pSS | 2b-d |
| 13 | 1 | 1 | **1** | 1 | 1 | 1 | 0 | 8 | pSS | 2e-g |
| 14 | 1 | 1 | **1** | 0 | 1 | 1 | ND | 5 | pSS | 2e-g |
| 15 | 1 | 1 | **1** | 1 | 1 | 0 | 1 | 7 | pSS | 2e-g |

**Table S2.** Clinical characteristics of patients donating biopsies for data presented in Figure 2a. Dry mouth complaints were defined as need to drink water during the night, Histopathology was considered positive if the focus score (identified as number of mononuclear infiltrates containing ≥50 lymphocytes/4 mm^2^ of glandular tissue) was ≥1 . Serum levels of anti-SSA/Ro and anti-SSB/La antibodies were assessed with ELISA tests. Unstimulated whole saliva (UWS) drop was evaluated by measuring the saliva production in 15 min. UWS ≤1.5 mL/15 min was considered abnormal. Ocular dryness assessed using both an abnormal ocular staining score (OSS) of ≥5 or a Schirmer’s test result of ≤ 5 mm/5 minutes. Ultrasound was scored using the Hocevar et al scoring system, as validated in Mossel *et al*, Annals Rheumatic Diseases, 2017. Classification was performed according to ACR-EULAR criteria and clinical characteristics presented. With a score of ≥ 4, the patient is classified as pSS. A score of ≤ 3 results in classification of incomplete pSS. Histopathology and autoantibody titre are weighted with 3 points each. ND = no data. ‘0’ and ‘1’ in table represent a negative or positive result for the relevant test, respectively.
